# Molecular subtyping of gastroesophageal dysplasia heterogeneity according to TCGA/ACRG classes

**DOI:** 10.1007/s00428-022-03392-7

**Published:** 2022-08-04

**Authors:** Valentina Angerilli, Gianmaria Pennelli, Francesca Galuppini, Stefano Realdon, Alberto Fantin, Edoardo Savarino, Fabio Farinati, Luca Mastracci, Claudio Luchini, Matteo Fassan

**Affiliations:** 1 Department of Medicine (DIMED), Surgical Pathology & Cytopathology Unit, University of Padua, via Gabelli 61, 35121 Padua, Italy; 2 Istituto Oncologico Veneto—IOV-IRCCS, Padua, Italy; 3 Department of Surgery, Oncology and Gastroenterology (DISCOG), University of Padua, Padua, Italy; 4 Department of Surgical Science and Integrated Diagnostics (DISC), University of Genova, Genoa, Italy; 5 Department of Pathology and Diagnostics, University and Hospital Trust of Verona, Verona, Italy

**Keywords:** Gastroesophageal dysplasia, Gastroesophageal cancer, Molecular classification

## Abstract

Gastric adenocarcinoma has recently been classified into several subtypes on the basis of molecular profiling, which has been successfully reproduced by immunohistochemistry (IHC) and in situ hybridization (ISH). A series of 73 gastroesophageal dysplastic lesions (37 gastric dysplasia and 36 Barrett dysplasia; 44 low-grade dysplasia and 29 high-grade dysplasia) was investigated for mismatch repair proteins, E-cadherin, p53, and EBER status, to reproduce The Cancer Genome Atlas (TCGA) and Asian Cancer Research Group (ACRG) molecular clustering. Overall, the dysplastic lesions were classified as follows: according to TCGA classification, EBV, 0/73 (0%), MSI, 6/73 (8.2%), GS, 4/73 (5.5%), CIN, 63/73 (86.3%); according to ACRG molecular subtyping, MSI, 6/73 (8.2%), MSS/EMT, 4/73 (5.5%), MSS/TP53^−^, 33/73 (45.2%), MSS/TP53^+^, 30/73 (41.1%). A positive association was found between MSS/TP53^−^ and Barrett dysplasia (*p* = 0.0004), between MSS/TP53^+^ and LG dysplasia (*p* = 0.001) and between MSS/TP53^+^ and gastric dysplasia (*p* = 0.0018). Gastroesophageal dysplastic lesions proved to be heterogenous in terms of TCGA/ACRG classes, but with a different distribution from that of cancers, with no EBV-positive cases, an increasing presence of mismatch repair deficiency from low grade to high grade lesions, and a prevalence of p53 aberrations in Barrett dysplasia. The present study further demonstrated that gastroesophageal dysplastic lesions may be characterized by alterations in predictive/prognostic biomarkers, and this should be considered in routine diagnostic.

## Introduction

Gastric and Gastroesophageal Junction (G/GEJ) adenocarcinomas represent a major global health burden and are among the leading causes of cancer-related deaths [1].

By the histopathological point of view, gastric adenocarcinomas have been traditionally divided into intestinal and diffuse subtypes according to the Lauren classification [2]. In 2019, the World Health Organization has proposed an alternative classification system, subdividing G/GEJ adenocarcinomas into different histological patterns comprising papillary, tubular, mucinous, and poorly cohesive carcinomas [3, 4]. However, histology-based classifications provide little clinical utility in the era of precision oncology. Great efforts have been made to dissect the molecular heterogeneity of gastroesophageal tumors and to develop novel classifications to better stratify patients and guide the therapeutic decision-making process.

Two landmark molecular classification systems have been published by The Cancer Genome Atlas (TCGA) [5] and by the Asian Cancer Research Group (ACRG) [6] by analyzing data from multiple platforms. TCGA study identified four genomic subtypes: chromosomal instability (CIN), microsatellite instability (MSI), genome stable (GS), and Epstein-Barr virus (EBV). The ACRG recognized four molecular subtypes: MSI, microsatellite stable/epithelial to mesenchymal transition (MSS/EMT), intact p53 activity (MSS/TP53^+^), and inactive p53 (MSS/TP53^−^) [7].

Comprehensive gene expression profiling has not extensively entered into clinical practice, and most available approaches on cancer RNA profiling are constrained by FFPE (formalin fixed and paraffin embedded tissue) related genetic material qualification issues [8]. On this ground, in 2016, Ahn and colleagues proposed a molecular classification of gastric cancer by using immunohistochemistry (IHC) and in in situ hybridization (ISH) and identified 5 clusters based on the sequential expression of EBER, MLH1, E-cadherin, and p53 [9].

Intestinal-type gastric cancer and Barrett’s adenocarcinoma develop through a multistep carcinogenetic cascade triggered primarily by longstanding inflammatory insults. The progressive phenotypic dedifferentiation which entails the progression from normal mucosa to intestinal metaplasia (Barrett’s esophagus or atrophic gastritis with intestinalized glands) to dysplasia and invasive carcinoma underlies a progressive accumulation of genetic alterations [10, 11]. Our group has already investigated the dysregulation of molecular biomarkers in gastroesophageal dysplastic lesions, including HER2 [12], PD-L1 [13], mismatch repair (MMR) status [13], and EBER [14].

The present study aimed to investigate the TCGA and ACRG subtype distribution across G/GEJ dysplastic lesions according to their differentiation profiles (i.e., low- versus high-grade lesions) and their anatomical location (i.e., gastric versus gastroesophageal junction).

## Materials and methods

### Case selection

A mono-institutional series of 73 FFPE tissue samples obtained from 73 Caucasian patients (M/F 49/24; mean age 64.3) were retrospectively selected from the archives of the Surgical Pathology Unit at Padua University. All information regarding human material was managed using anonymous numerical codes, and all samples were handled in compliance with the Declaration of Helsinki (https://www.wma.net/what-we-do/medical-ethics/declaration-of-helsinki/).

The series included 65 endoscopy biopsies (36 gastric and 29 Barrett’s esophagus-related dysplasia) and 8 endoscopic resections (5 gastric and 3 Barrett’s esophagus-related dysplasia). Original slides were jointly re-evaluated by two GI pathologists (MF and GP).

### Immunohistochemistry

IHC was performed using the Bond Polymer Refine Detection kit (Leica Biosystems, Newcastle upon Tyne, UK) in the BOND-MAX system (Leica Biosystems). Four-micrometer-thick FFPE sections were incubated with the following primary antibodies: PMS2 (clone EP51; dilution: 1:50 Dako), MSH6 (clone EP49; dilution: 1:50; Dako), E-cadherin (clone NCH-38; dilution: 1:50; Dako), and p53 (clone DO-7; dilution: 1:150; Dako). IHC slides were jointly evaluated by three pathologists (VA, GP, and MF).

Mismatch Repair defective status was assessed by testing PMS2 and MSH6, and samples were defined as dMMR when one or both proteins resulted negative [15]. In case of protein loss, the dominant component of the heterodimer (i.e., MLH1 for PMS2 and MSH2 for MSH6; Dako) was tested.

p53 was considered as aberrant in the presence of complete loss or diffuse and strong nuclear immunostaining in dysplastic cells [9, 16].

E-cadherin expression was considered altered in the presence of complete loss or markedly reduced membranous staining (> 30%), regardless of nuclear/cytoplasmic staining [9].

### *EBER *in situ* hybridization*

The Bond ready-to-use ISH EBER Probe was used in a Leica Bond-Max automation system according to the manufacturer’s instructions (Leica Biosystems) to detect EBV infection.

### Statistical analysis

Chi-square and Fisher’s exact tests were tested, where appropriate. *p* values < 0.05 were considered statistically significant.

## Results

### Histomorphologic characterization of the series

Out of the 73 samples, 37 were gastric dysplastic lesions (24 low-grade [LG] and 13 high-grade [HG]), and 36 were gastroesophageal Barrett-associated dysplastic lesions (20 LG and 16 HG). Of the 37 gastric cases, 35 were from antral/angular zone and 2 from the oxyntic compartment. Of the 36 Barrett-related cases, 19 were from the distal esophagus (Siewert type 1), and 17 were from the GEJ (Siewert type 2).

Among the gastric cases, 30/37 (81.1%) were of intestinal type (two of them cryptic subtype), 6/37 (16.2%) were of foveolar type, and 1/37 (2.7%) was of mixed. Of note, four out of 37 gastric dysplastic cases were adenomas, three foveolar adenomas, and one intestinal adenoma. Among the Barrett-related cases, 33/36 (91.7%) were of intestinal type, 2/36 (5.6%) were of foveolar type, and 1/36 (2.8%) was of mixed type.

### Biomarkers’ alterations distribution within dysplastic lesions

**Fig. 1:**
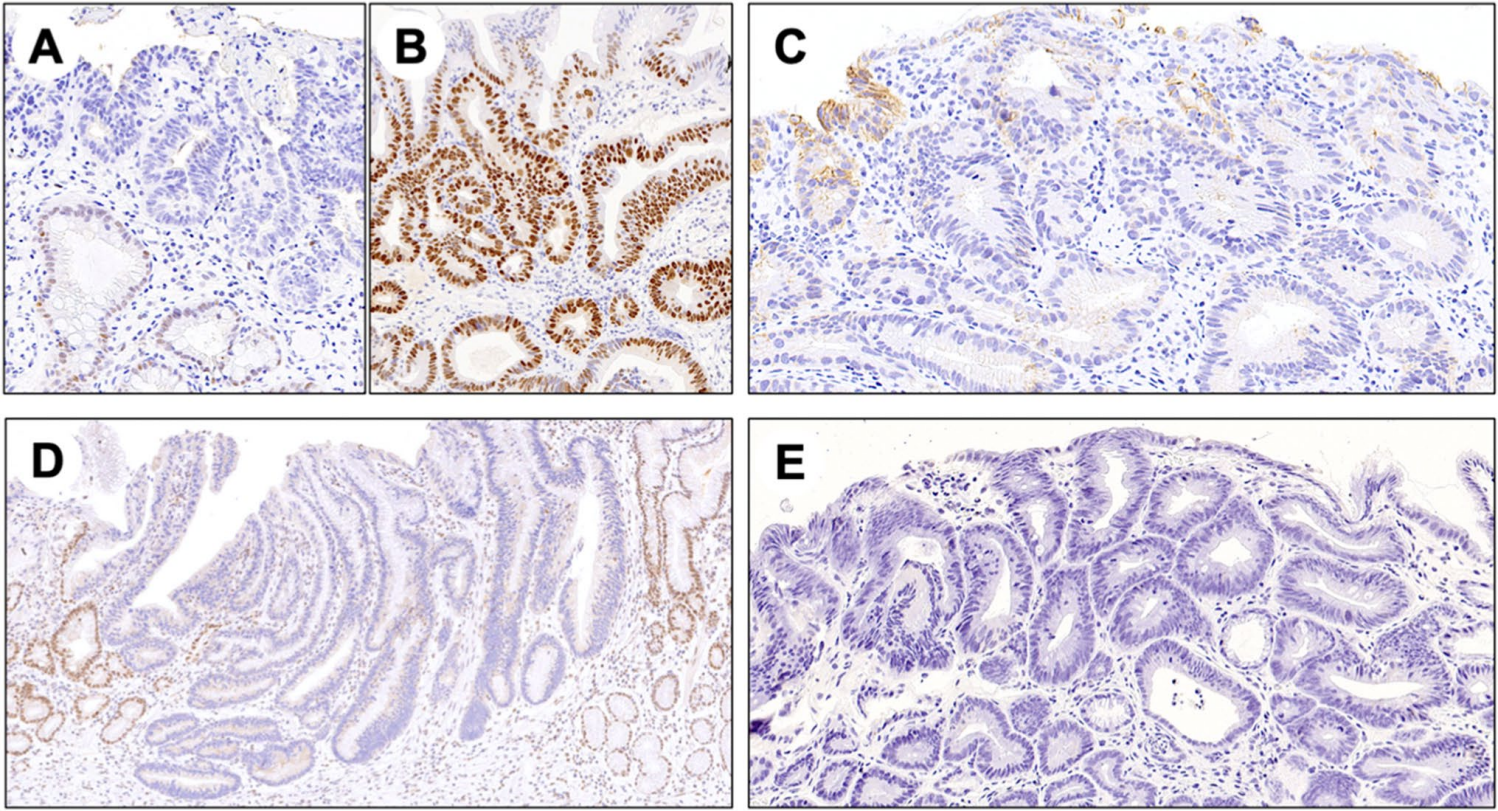
Representative samples of biomarkers status in gastric and Barrett-related dysplastic lesion. (**A**) A p53 positive (loss pattern) Barrett dysplasia. (**B**) A p53 positive (clonal pattern) Barrett dysplasia. (**C**) An E-cadherin negative Barrett dysplasia. (**D**) A PMS2 negative gastric dysplasia in a surgical specimen sample. (**E**) An EBER-negative gastric dysplasia

The detailed distribution of the obtained results according to the single tested biomarker is summarized in (Table 1)*.* In this study, we analyzed the expression of PMS2, MSH6, E-cadherin, p53, and EBER in 73 gastroesophageal dysplastic lesions (Fig. 1). Out of the 73 samples, 37 were gastric dysplastic lesions (24 low-grade [LG] and 13 high-grade [HG]), and 36 were gastroesophageal Barrett-associated dysplastic lesions (20 LG and 16 HG). Of the 37 gastric cases, 35 were from antral/angular zone and 2 from the oxyntic compartment. Of the 36 Barrett-related cases, 19 were from the distal esophagus (Siewert type 1), and 17 were from the GEJ (Siewert type 2).

**Table 1:** PMS2, MSH6, E-cadherin, p53, and EBER expression status in low and high-grade gastric and Barrett-related gastroesophageal junction dysplastic lesions

		*Gastric* *(n* = *37)*			*Gastroesophageal junction* *(n* = *36)*		
		LG (*n* = 24)	HG (*n* = 13)	Total	LG (*n* = 20)	HG (*n* = 16)	Total
PMS2	Retained	23 (95.8%)	9 (69.2%)	32 (86.5%)	19 (95.0%)	16 (100%)	35 (97.2%)
	Loss	1 (4.2%)	4 (30.8%)	5 (13.5%)	1 (5.0%)	0 (0.0%)	1 (2.8%)
MSH6	Retained	24 (100%)	13 (100%)	37 (100%)	20 (100%)	16 (100%)	36 (100%)
	Loss	0 (0.0%)	0 (0.0%)	0 (0.0%)	0 (0.0%)	0 (0.0%)	0 (0.0%)
E-cadherin	Retained	24 (100%)	12 (76.9%)	36 (97.3%)	19 (95.0%)	14 (87.5%)	33 (83.3%)
	Loss	0 (0.0%)	1 (23.1%)	1 (2.7%)	1 (5.0%)	2 (12.5%)	3 (16.7%)
p53	Aberrant	3 (12.5%)	6 (46.2%)	9 (24.3%)	13 (65.0%)	12 (75.0%)	25 (69.4%)
	wt	21 (87.5%)	7 (53.8%)	28 (75.7%)	7 (35.0%)	4 (25.0%)	11 (30.6%)
EBER	pos	0 (0.0%)	0 (0.0%)	0 (0.0%)	0 (0.0%)	0 (0.0%)	0 (0.0%)
	neg	24 (100%)	13 (100%)	37 (100%)	20 (100%)	16 (100%)	36 (100%)

*pos* positive, *neg* negative, *wt* wild type, *n* number, *LG* low grade, *HG* high grade

Mismatch repair deficiency, defined by the loss of PMS and/or MSH6, was detected in 5/37 (13.5%) of gastric dysplasia and 1/36 (2.8%) of Barrett dysplasia; in 2/44 (4.5%) of LG dysplasia and in 4/29 (13.8%) of HG dysplasia; in 1/25 (4.0%) of LG gastric dysplasia, 4/13 (30.8%) of HG gastric dysplasia, 1/20 (5.0%) of LG Barrett dysplasia and was absent in HG Barrett dysplasia.

E-cadherin was altered in 1/37 (2.7%) of gastric dysplasia and in 3/36 (8.3%) of Barrett dysplasia; in 1/44 (2.3%) of LG dysplasia and in 3/29 (10.3%) of HG dysplasia; in 1/13 (7.7%) of HG gastric dysplasia, 1/20 (5.0%) of LG Barrett dysplasia, 2/16 (12.5%) of HG Barrett and was retained in all the cases of LG gastric dysplasia.

p53 had an aberrant phenotype in 9/37 (24.3%) of gastric dysplasia and in 25/36 (69.4%) of Barrett dysplasia; in 16/44 (36.3%) of LG dysplasia and in 18/29 (62.1%) of HG dysplasia; in 3/24 (12.5%) of LG gastric dysplasia, 6/13 (46.2%) of HG gastric dysplasia, 13/20 (65.0%) of LG Barrett dysplasia and 12/16 (75.0%) of HG Barrett dysplasia.

EBV RNA (EBER) was not detected in any of the tested dysplastic lesion.

A positive association was found between aberrant p53 phenotype and HG gastric dysplasia (*p* = 0.0425) and between aberrant p53 phenotype and Barrett dysplasia (0.0002).

### Molecular subtypes of gastroesophageal dysplasia

By following the taxonomic sequence proposed by Ahn and colleagues [9], we classified the 73 gastroesophageal dysplastic lesions into TCGA and ACRG molecular subtypes. Additionally, we classified our samples into the five clusters identified by Ahn and colleagues [9].

Overall, our samples were classified as follows: according to TCGA classification, EBV, 0/73 (0%), MSI, 6/73 (8.2%), GS, 4/73 (5.5%), CIN, 63/73 (86.3%); according to ACRG molecular subtyping, MSI, 6/73 (8.2%), MSS/EMT, 4/73 (5.5%), MSS/TP53^−^, 33/73 (45.2%), MSS/TP53^+^, 30/73 (41.1%); according to Ahn’s clustering: C1 (EBV tumors), 0/73 (0%), C2 (MSI tumors), 6/73 (8.2%), C3 (EMT tumors), 4/73 (5.5%), C4 (aberrant p53 expression), 33/73 (45.2%), and C5 (normal p53 expression), 30/73 (41.1%).

According to TCGA classification, dysplastic lesions were subdivided as follows: gastric dysplasia: EBV, 0/37 (0.0%), MSI, 5/37 (13.5%), GS, 1/37 (2.7%), and CIN, 31/37 (83.8%); Barrett dysplasia: EBV, 0/36 (0.0%), MSI, 1/36 (2.8%), GS, 3/36 (8.3%), and CIN, 32/36 (88.9%); LG dysplasia: EBV, 0/44 (0.0%), MSI, 2/44 (4.5%), GS, 1/44 (2.3%), and CIN, 41/44 (93.2%); HG dysplasia. EBV, 0/29 (0%), MSI, 4/29 (13.8%), GS, 3/29 (10.3%), and CIN, 22/29 (75.9%).

No statically significant association was observed between TCGA subtypes and dysplasia subcategory.

According to ACRG classification, dysplastic lesions were categorized as follows: gastric dysplasia: MSI, 5/37 (13.5%), MSS/EMT, 1/37 (2.7%), MSS/TP53^−^, 9/37 (24.3%), MSS/TP53^+^, 22/37 (59.5%); Barrett dysplasia: MSI, 1/36 (2.8%), MSS/EMT, 3/36 (8.3%), MSS/TP53^−^, 24/36 (66.7%), MSS/TP53^+^, 8/36 (22.2%); LG dysplasia: MSI, 2/44 (4.5%), MSS/EMT, 1/44 (2.3%), MSS/TP53^−^, 16/44 (36.4%), MSS/TP53^+^, 25/44 (56.8%); HG dysplasia: MSI, 4/29 (13.8%), MSS/EMT, 3/29 (10.3%), MSS/TP53^−^, 17/29 (58.6%), MSS/TP53^+^, 5/29 (17.2%).

A positive association was found between MSS/TP53^−^ and Barrett dysplasia (*p* = 0.0004), between MSS/TP53^+^ and LG dysplasia (*p* = 0.001), and between MSS/TP53^+^ and gastric dysplasia (*p* = 0.0018).

According to Ahn’s clustering, dysplastic lesions were subdivided as follows: gastric dysplasia, C1, 0/37 (0.0%), C2, 5/37 (13.5%), C3, 1/37 (2.7%), C4, 9/37 (24.3%), C5, 22/37 (59.5%); Barrett dysplasia: C1, 0/36 (0.0%), C2, 1/36 (2.8%), C3, 3/36 (8.3%), C4, 24/36 (66.7%), C5, 8/36 (22.2%); LG dysplasia, C1, 0/44 (0.0%), C2, 2/44 (4.5%), C3, 1/44 (2.3%), C4, 16/44 (36.4%), C5, 25/44 (56.8%); HG dysplasia, C1, 0/29 (0%), C2, 4/29 (13.8%), C3, 3/29 (10.3%), C4, 17/29 (58.6%), C5, 5/29 (17.2%).

A positive association was found between C4 and Barrett dysplasia (*p* = 0.0004), between C5 and LG dysplasia (*p* = 0.001), and between C5 and gastric dysplasia (*p* = 0.0018).

**Fig. 2:**
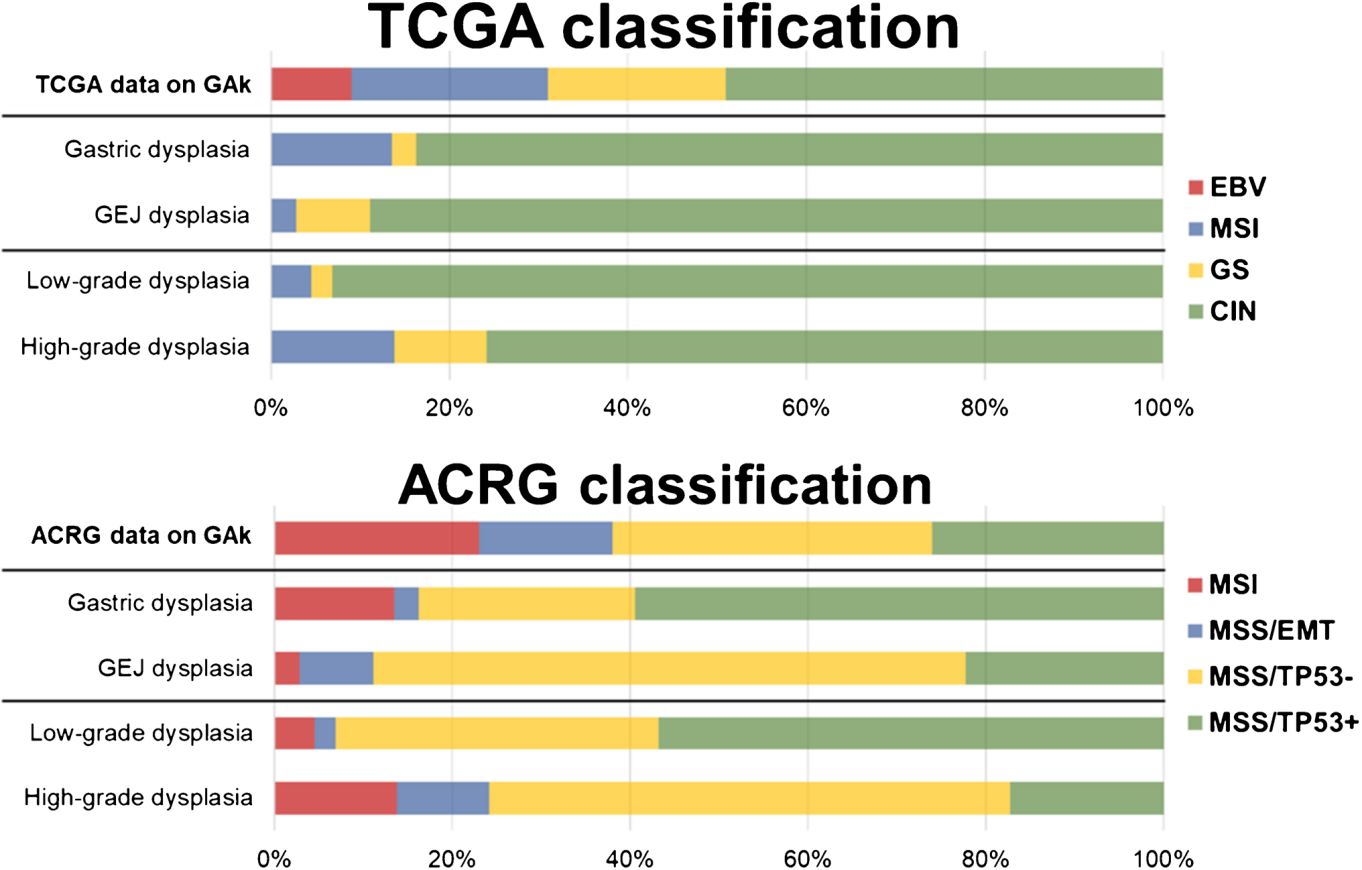
Graphic representation of the distribution of our samples (gastric LG, gastric HG, gastroesophageal junction LG, and gastroesophageal junction HG dysplastic lesions) across TCGA and ACRG classifications

The distribution of the molecular subtypes across gastric and Barrett and LG and HG dysplastic lesions is summarized in Fig. 2.

No correlation was found between molecular subtype and histological dysplasia type (i.e., intestinal, foveolar, and mixed). Among the foveolar cases, 6/8 were MSS/TP53 + /CIN, 1/8 was MSS/TP53^−^/CIN, and 1/8 was MSI. The two mixed cases were MSS/TP53^−^/CIN. The two cryptic cases were MSS/TP53 + /CIN.

## Discussion

Previous works [7, 14, 15] have conducted biomarker expression-based subtyping of gastric adenocarcinomas based on the sequential expression of EBER, MLH1, E-cadherin, and p53 and found correspondence with TCGA and ACRG molecular classifications. However, there is no available data in literature regarding gastroesophageal precursor lesions.

The present study provides a molecular classification of gastroesophageal dysplastic lesions accomplished by using IHC and ISH.

The first striking difference that emerges when applying molecular subtyping of gastric adenocarcinoma to our cohort is the absence of EBV-associated dysplastic lesions. According to TGCA network, EBV-associated gastric cancers account for 9% of gastric cancers world-wide [5]. However, reports of the prevalence of EBV-associated gastric carcinoma have ranged from 1.3 to 20.1% in different countries [17]. EBV promotes carcinogenesis through DNA methylation of a series of tumor suppressor genes, resulting in uncontrolled cell growth and though the creation of a pro-inflammatory environment [18]. How and when EBV gets into gastric epithelial cells during gastric carcinogenesis remain unclear. In agreement with our results, many studies have failed to detect EBV in gastroesophageal precursor lesions, suggesting that EBV infection is a late event in gastric carcinogenesis[19, 20]. Of note, an EBV-associated gastric dysplasia adjacent to an EBV-positive gastric adenocarcinoma has been previously described by our group as an exceptionally rare finding [14].

MSI/dMMR identifies a subtype of gastroesophageal adenocarcinomas with specific clinicopathologic and molecular features which can benefit from treatment with immune checkpoint inhibitors. According to TCGA and ACRG data, MSI gastric cancers represent 22–23% of all gastric cancers. Overall, we found a prevalence of MSI/dMMR dysplastic lesions of 8.2%. Loss of MLH1 and PMS2 was the only pattern of dMMR detected in our series.

Variability among different ethnicities and inconsistency of histological criterial and detection methods render the comparison between our results and available data particularly difficult.

Our results point out that MSI/dMMR is part of the plethora of molecular alterations in gastroesophageal dysplasia and may be considered an early event in the carcinogenetic process. In our series, the prevalence of dMMR was higher in HG than LG-dysplasia overall, supporting the hypothesis that more advanced lesions are the result of an increase in genetic and molecular alterations [13, 21]. Unsurprisingly, the prevalence of dMMR was higher in gastric than Barrett dysplasia. According to TCGA genomic characterization, GEJ adenocarcinomas have a lower prevalence of MSI, with no MSI tumors of clear esophageal origin identified. It is well-known that dMMR and clonal *p53* can coexist; however, none of samples had a concomitant altered MMR and p53 status.

The cluster identified by the loss of expression of E-cadherin corresponds to the GS subtype (~ 20%) or the MSS/EMT subtype (~ 15%). It must be noted that GS and MSS/EMT subtypes are highly enriched in Lauren diffuse gastric adenocarcinoma. In fact, according to Birkman and colleagues, 51.0% of diffuse-type tumors had aberrant E-cadherin expression versus 1.6% of intestinal-type tumors [22]. Evidence suggests that sporadic diffuse gastric cancer may develop from an “alternative” carcinogenetic pathway (i.e., alternative to the dysplasia-invasive carcinoma pathway of Lauren intestinal type) in the context of atrophic/metaplastic gastritis triggered by *CDH1* loss of function [23]. However, several *CDH1* inactivation mechanisms (promoter methylation, microRNAs post-transcriptional regulation, glycosylation) can be associated with Lauren intestinal-type gastric adenocarcinoma and can also be present in early stages of neoplastic development, as shown by our results [24].

The use of E-cadherin to establish the GS and EMT/GS subtypes in gastroesophageal precursor lesions and adenocarcinomas has several limitations. First, the relation between E-cadherin altered expression and *CDH1* loss of function is controversial [25]. Additionally, GS and MSS/EMT subtypes are enriched in *RHOA* mutations which would not be detected.

In our cohort, most cases formed the cluster with aberrant p53 expression, which corresponds to the CIN (~ 50%) and MSS/TP53^−^ (~ 36%) subtypes. The rate of aberrant p53 expression increased from LG to HG dysplasia, further indicating that *TP53* alterations are early drivers of carcinogenesis and can also contribute to its progression. Furthermore, Barrett dysplastic lesions showed a higher prevalence of aberrant p53. As previously described, p53 overexpression is part of the spectrum of molecular alterations of Barrett’s esophagus and is significantly associated with increased risk of neoplastic progression [26].

Interestingly, a recent work by Flinner and colleagues [27] found by comparison with OncoScan data that the distinction between GS and CIN based on E-cadherin and p53 status and Lauren morphology can lead to mislabeling. In this context, the use of microsatellite-based multiplex PCR assay for the detection of allelic imbalance or loss of heterozygosity has proved to be a simple, quantitative, and easily interpretable surrogate to identify CIN and to enable the differentiation of GS and CIN [28].

A growing body of evidence indicates that G/GEJ cancers should be considered as a molecular spectrum, with a progressive increase of chromosomal instability phenotype proximally [29]. A limit of the study is to have considered a mixture of Siewert type I and type II among Barrett dysplasia and of antral and corpus dysplasia, to have a relatively large number of cases. Further larger series should focus on the topographic-related molecular landscape of G/GEJ dysplastic lesions.

Overall, our data further pinpointed that gastric and gastroesophageal preinvasive lesions may be characterized by alterations in predictive/prognostic biomarkers. This should be considered when assessing biomarkers’ status in those biopsy samples in which both dysplasia and invasive adenocarcinoma are present. Thus, an incorrect morphologic distinction between pre-invasive lesion and adenocarcinoma may hamper an accurate biomarkers’ status assessment [30].

In conclusion, the present study demonstrated that a simple IHC/ISH-based classification can be applied to gastroesophageal precursor lesions. Gastroesophageal dysplastic lesions have proved to be heterogenous in terms of TCGA/ACRG classes, but with a different distribution in comparison with cancers, shedding light into the biology of tumors arising in such district.
